# Cost-Effectiveness of Biomarker-Associated Early Pancreatic Cancer Detection in New-Onset Diabetes

**DOI:** 10.1001/jamanetworkopen.2025.38031

**Published:** 2025-10-17

**Authors:** Irena Stefanova, Nathan Thompson, Lucy Oldfield, Martyn Stott, Robert Hanson, Daniel Palmer, Christopher Halloran, William Greenhalf, Robert Van Der Meer, Eithne Costello

**Affiliations:** 1 Department of Molecular and Clinical Cancer Medicine, University of Liverpool, Liverpool, United Kingdom; 2 Department of Pancreas Surgery, Royal Liverpool Hospital NHS Foundation Trust, Liverpool, United Kingdom; 3 Management Science, University of Strathclyde, Glasgow, Scotland, United Kingdom; 4 Liverpool Clinical Trials Centre, University of Liverpool, Liverpool, United Kingdom; 5 Clatterbridge Cancer Centre, Liverpool, United Kingdom

## Abstract

**Importance:**

Pancreatic ductal adenocarcinoma (PDAC) is often diagnosed late, resulting in a dismal survival. Only 1 in 5 patients is eligible for potentially curative surgery. Although earlier detection of PDAC could significantly improve prognosis, population-wide screening is not currently feasible. Individuals at high risk, such as those with new-onset diabetes, among whom approximately 10% have type 3c diabetes (T3cD) and 1% have undiagnosed PDAC, could represent a target population for PDAC screening.

**Objective:**

To compare the cost-effectiveness of 3 PDAC screening strategies in new-onset diabetes with the standard of care.

**Design, Setting, and Participants:**

For this economic evaluation, a Markov state-transition decision model was developed to assess the downstaging benefits associated with screening policies over a 5-year period. The model allowed for diagnosis of resectable, borderline resectable, locally advanced, or metastatic disease. Model parameters were subject to 1-way and multiway sensitivity analyses. Mean treatment costs were extracted from the UK National Healthcare Service cost-collection data (2021-2022), and the screening strategies were applied to a simulated cohort of individuals aged 50 years or older with new-onset diabetes in the UK. Analyses were performed from August 2024 to January 2025.

**Exposures:**

Pancreatic ductal adenocarcinoma screening strategies included the use of a T3cD biomarker, a cancer-specific biomarker, or a combination of a T3cD biomarker followed by a cancer-specific test in the population with new-onset diabetes. Biomarker cohorts were compared with the standard-of-care cohort undergoing diagnostic investigations on the development of clinical symptoms.

**Main Outcomes and Measures:**

Per-person incremental cost-effectiveness ratios (ICERs) and net benefits were calculated, allowing for a willingness-to-pay threshold per quality-adjusted life-year (QALY) of £30 000 (US $40 156), as accepted by the UK’s National Institute for Health and Care Excellence.

**Results:**

Among individuals aged 50 years or older with new-onset diabetes, neither the cancer-specific nor T3cD biomarker strategy was cost-effective compared with the standard of care, with an ICER per QALY of £71 906 (US $96 249) for the cancer-specific biomarker test and an ICER per QALY of £46 371 (US $62 070) for the T3cD biomarker strategy. Sequential use of a T3cD biomarker and a cancer-specific biomarker approached cost-effectiveness (ICER per QALY, £34 223 [US $45 809]), with 2.4 scans performed to detect 1 PDAC case among 200 000 people with new-onset diabetes. Sensitivity analyses highlighted biomarker specificity as a critical determinant of cost-effectiveness.

**Conclusions and Relevance:**

In this economic evaluation of early PDAC detection among individuals with new-onset diabetes, the application of a primary T3cD biomarker followed by a secondary PDAC-specific test was more cost-effective than the sole use of either biomarker, although no strategy was cost-effective compared with standard of care. These findings can be used to inform biomarker-associated early detection strategies for PDAC.

## Key Points

**Question:**

What is the most cost-effective biomarker-associated pancreatic ductal adenocarcinoma (PDAC) screening strategy for individuals with new-onset diabetes?

**Findings:**

In this economic evaluation of early PDAC detection in new-onset diabetes, the sequential use of a type 3c diabetes (T3cD) biomarker test and a cancer-specific biomarker test approached cost-effectiveness (incremental cost-effectiveness ratio per quality-adjusted life-year of £34 223 [US $45 809]) compared with the current standard of care, where no routine screening is applied.

**Meaning:**

This study suggests that, given the characteristics, particularly the specificity, of published biomarkers, screening of the high-risk group of individuals with new-onset diabetes for PDAC approaches cost-effectiveness when biomarker tests first enable enrichment for T3cD and then select for PDAC in this enriched group.

## Teaser

This economic evaluation examines the cost-effectiveness of 3 screening strategies for pancreatic ductal adenocarcinoma among individuals with new-onset diabetes and compares them with the standard of care.

## Introduction

Pancreatic ductal adenocarcinoma (PDAC) has a low overall 5-year survival rate (8.3% in England).^1^ Over half of patients with PDAC present with distant metastases, with a median survival of 4.8 months (range, 2.9-6.6 months).^2^ By contrast, those with resectable disease undergoing potentially curative surgery survive up to 35 to 54.4 months.^3^ Earlier detection, increasing eligibility for surgery, could significantly improve prognosis.

Population-wide screening for PDAC is currently unfeasible^4^ due to the low incidence of PDAC (17.2 per 100 000 people)^5^ and the lack of highly accurate screening tests. To avoid large numbers of false-positive results and the ensuing distress or harm from overdiagnosis and overtreatment, a cancer detection test aimed at an average-risk population needs to be extremely specific. Therefore, PDAC screening is recommended only for high-risk groups, including individuals with familial predisposition, specific genetic syndromes, and other nongenetic risk factors.^6^ For a screening program to be ethical and effective, it must meet defined criteria,^7^ including the use of a test that is noninvasive, low cost, and acceptable to the target population. Economic evaluation should be undertaken to assess its value from a health care perspective. The prospective multicenter cohort study Cancer of Pancreas Screening (CAPS) demonstrated that surveillance strategies in specific high-risk groups, such as individuals with familial or inherited risk of PDAC, resulted in earlier cancer detection with a notable shift in detected disease stage toward an increased proportion of resectable cases.^8^ Within the CAPS cohort, 57.9% of patients diagnosed via surveillance had stage I disease, while 5.2% of patients received a diagnosis of stage IV disease.^8^

Approximately 85% of patients with PDAC have impaired glucose regulation at the time of cancer diagnosis, with 40% to 65% experiencing diabetes.^9^ Diabetes among these patients is frequently of recent onset, occurring within 3 years of cancer diagnosis,^10,11,12^ and the risk of developing PDAC for individuals aged 50 years or older with new-onset diabetes is 6-fold to 8-fold higher than among the general population.^10,13,14^ Hence, new-onset diabetes in this age group may be an early warning sign of PDAC and a trigger for screening, along with other risk factors.

Approximately 10% of type 2 diabetes cases arise secondary to a disease of the pancreas gland (type 3c diabetes [T3cD]), although it is often misdiagnosed as type 2 diabetes.^15^ Pancreatic ductal adenocarcinoma–related diabetes comprises approximately 10% of T3cD cases, with chronic pancreatitis accounting for approximately 80% of cases.^15^ Identifying T3cD among individuals with new type 2 diabetes cases would help stratify individuals with new-onset diabetes at high risk for PDAC screening. At present, there is no clinically used test for T3cD, although candidate markers are being explored.^16^ To advance the development of early PDAC biomarkers in new-onset diabetes, Cancer Research UK funded the United Kingdom Early Detection Initiative (UK-EDI), a national prospective cohort study recruiting individuals aged 50 years or older with new-onset diabetes, collecting longitudinal clinical information and biospecimens for the development of molecular, epidemiologic, and demographic PDAC biomarkers.^17^

As a component of UK-EDI, this study investigates the cost-effectiveness of using a T3cD biomarker test, a cancer-specific biomarker test, or a combination of the 2 tests to screen individuals aged 50 years or older with new-onset diabetes for PDAC compared with the standard of care, in which no routine screening is performed and patients are diagnosed after development of symptoms.

## Methods

### Study Design

In this economic evaluation, an integrated decision tree and a Markov state-transition model were developed to compare the cost-effectiveness of biomarker-associated screening for PDAC among individuals with new-onset diabetes compared with the standard of care for PDAC diagnosis and treatment. New-onset diabetes was defined as diabetes occurring among individuals aged 50 years or older within 6 months of diabetes diagnosis (hemoglobin A_1c_ level ≥48 mmol/mol [≥6.5%]; to convert to proportion of total hemoglobin, multiply by 0.01), in line with the National Institute for Health and Care Excellence (NICE) guidelines,^18^ as described in the UK-EDI study protocol.^17^ The UK-EDI Study^17^ received ethical approval from the London-West London and Gene Therapy Advisory Committee Research Ethics Committee. Considering the nature of this economic evaluation used a simulated population with new-onset diabetes, obtaining informed consent from study participants was not possible. This study was conducted and reported in accordance with the Consolidated Health Economic Evaluation Reporting Standards (CHEERS) reporting guideline.

**Figure 1.**
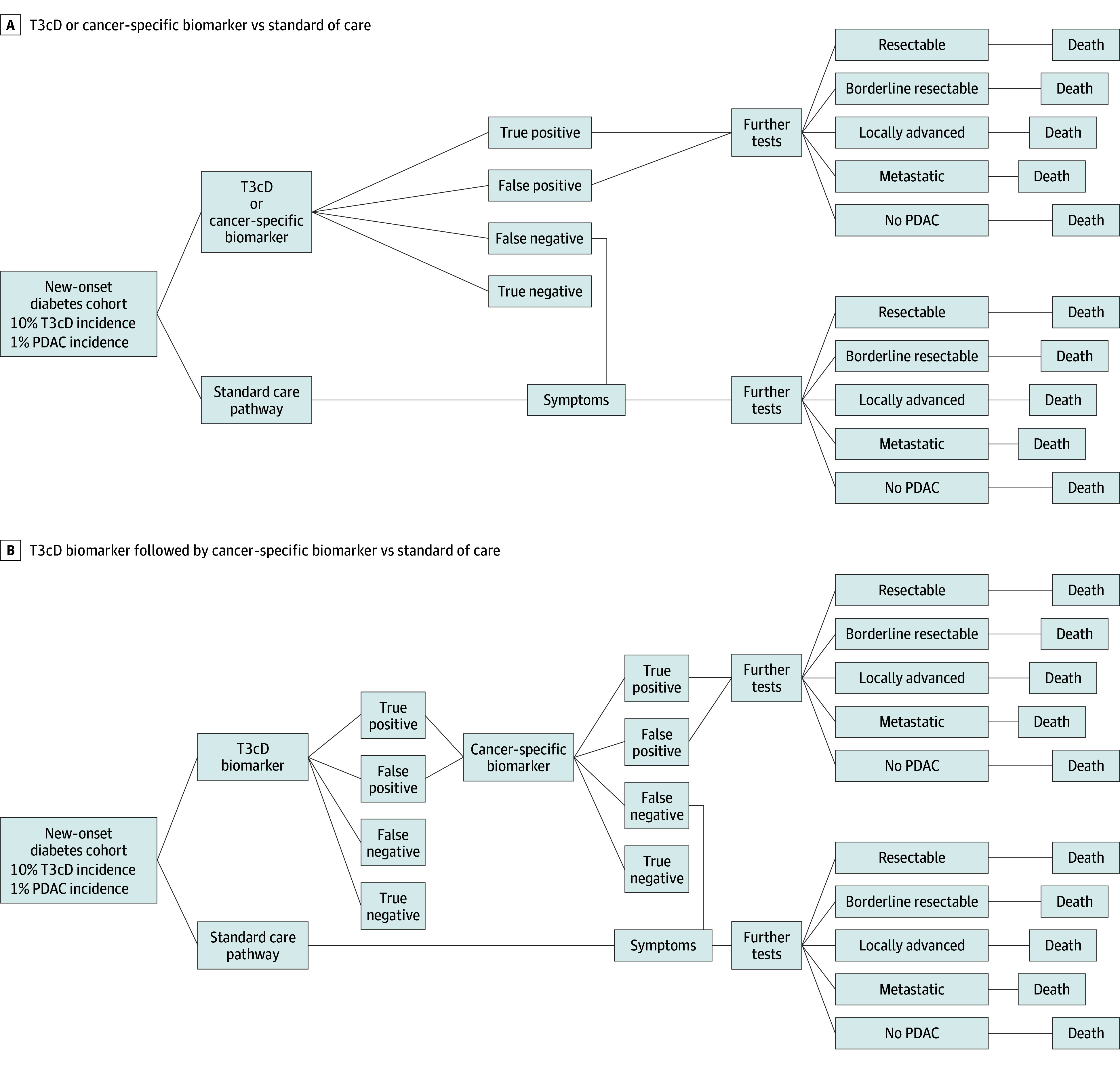
Markov State-Transition Model A, Application of a type 3c diabetes (T3cD) biomarker or a cancer-specific biomarker compared with the standard care pathway in the population with new-onset diabetes with a T3cD incidence of 10% and pancreatic ductal adenocarcinoma (PDAC) incidence of 1%. B, Application of a T3cD biomarker followed by a cancer-specific biomarker compared with the standard care pathway in the population with new-onset diabetes with a T3cD incidence of 10% in new-onset diabetes and PDAC incidence of 10% in T3cD. Further tests include computed tomography, endoscopic ultrasonography, and positron emission tomography; transition probabilities: resectable = 0.17, 3.7 years to death; borderline resectable = 0.39, 1.4 years to death; locally advanced = 0.41, 1.3 years to death; metastatic = 0.82, 0.4 years to death.

Entry into the pathway for the standard-of-care cohort occurred on the development of clinical symptoms suspicious of PDAC. Patients were subjected to routine diagnostic investigations, currently used for PDAC diagnosis, such as laboratory tests (carbohydrate antigen 19-9 [CA19-9]); computed tomography scan of thorax, abdomen, and pelvis; endoscopic ultrasonography; and positron emission tomography. For the biomarker-associated early detection strategies, the model considered the same diagnostic investigations. However, they were applied after a positive biomarker test result. All biomarker-based strategies were compared with the standard of care. The model allowed for the diagnosis of resectable, borderline resectable, locally advanced, or metastatic disease in all pathways. The proportion of patients at each disease stage was potentially influenced by early cancer detection, with an increase in diagnoses for patients with resectable tumors and a reduction in metastatic cancers in the biomarker strategies compared with the standard-of-care cohort (Table 1). Clinical and economical outcomes in each PDAC stage were tracked over a 5-year time horizon, or until death, with a cycle length of 1 year (Figure 1A).

**Table 1.** Markov State-Transition Model Input Parameters^a^

Model input parameter	Base case (range)
Treatment costs–biomarker pathway, £	
Resectable	47 581 (40 856-55 091)
Borderline resectable	37 187 (31 088-43 939)
Locally advanced	33 398 (29 369-37 687)
Metastatic	28 418 (24 802-33 231)
Treatment costs–standard pathway, £	
Resectable	44 203 (37 815-51 375)
Borderline resectable	31 947 (27 217-37 143)
Locally advanced	28 505 (25 855-31 217)
Metastatic	28 418 (24 802-32 231)
Diagnostic tests	
Biomarker cost, £	45 (20-100)
Biomarker pathway	2363 (2289-2438)
Standard care pathway	3087 (3068-3106)
Health utilities	
Healthy	0.86 (0.85-0.87)
Resectable	0.83 (0.82-0.85)
Borderline resectable	0.81 (0.81-0.81)
Locally advanced	0.80 (0.78-0.81)
Metastatic	0.76 (0.75-0.78)
Death	0 (0-0)
T3cD biomarker performance	
Sensitivity	0.80 (0.70-0.99)
Specificity	0.98 (0.70-0.99)
Cancer-specific biomarker performance	
Sensitivity	0.32 (0.30-0.70)
Specificity	0.95 (0.70-0.99)
Incidence, %	
T3cD in new-onset diabetes	10 (5-15)
PDAC in new-onset diabetes	1.0 (0.8-1.2)
PDAC in the general population	0.02 (0.01-0.03)
Discount, %	3.5 (3-4)
Starting state proportions, %	
Resectable	From 10 to 40 (5-15 to 35-45)
Borderline resectable	From 15 to 25 (10-20 to 20-30)
Locally advanced	From 20 to 25 (15-25 to 20-30)
Metastatic	From 55 to 10 (50-60 to 5-15)
Median survival, y	
Resectable	3.7 (2.9-4.5)
Borderline resectable	1.4 (1.0-1.8)
Locally advanced	1.3 (0.8-1.8)
Metastatic	0.4 (0.2-0.6)

Abbreviations: PDAC, pancreatic ductal adenocarcinoma; T3cD, type3c diabetes.

^a^ The conversion rate was £1 = US $1.34.

To evaluate the cost-effectiveness of a combination biomarker-associated screening, a 2-stage approach was taken. The first stage included application of a T3cD biomarker test to preselect the 10% of individuals with T3cD from among those with new-onset diabetes. To this preselected population of individuals with T3cD, a cancer-specific biomarker test was applied; on a positive result, patients underwent diagnostic investigations and treatment for PDAC (Figure 1B).

### Model Assumptions

The model assumed successful pancreatic resection in 59.3% of patients with borderline resectable disease^19^ and 8% of patients with locally advanced disease.^20^ For patients undergoing surgery, the estimated intensive care stay was 48 hours, and the mean length of hospital stay was 10 to 12 days, consistent with local hospital practice. In the group with metastatic disease, 60% were considered candidates for palliative chemotherapy, and 40% received palliative care only (based on local hospital data). In the cohorts with locally advanced and metastatic disease, the model allowed 84.4% of patients to undergo endoscopic retrograde cholangiopancreatography and biliary stent and 15.6% of patients to undergo percutaneous transhepatic cholangiography and stent.^21^ In the biomarker test–driven pathways, all patients with resectable disease were asymptomatic at diagnosis and assumed not to require preoperative biliary drainage, while in the standard-of-care pathway, 58% of patients were assigned to endoscopic retrograde cholangiopancreatography and biliary stent.^22^

Pancreatic consultant and specialist nurse reviews included a single appointment preoperatively and every 3 to 6 months postoperatively for patients with resectable, borderline resectable, or locally advanced disease undergoing pancreatic resection. Oncology consultant reviews included 2 appointments prior to commencing systemic chemotherapy and monthly thereafter. In the resectable disease stage only, adjuvant chemotherapy was considered in the models’ cost calculations, while for borderline resectable and locally advanced cancers, both induction and adjuvant chemotherapy were costed. Pancreatic enzyme supplements and diabetes care expenditures were accounted for from PDAC diagnosis until death based on median overall survival.

### Biomarker Performance and Cost

There are no biomarkers in routine clinical use for distinguishing T3cD from type 2 diabetes. The T3cD biomarker test pathway therefore used conservative biomarker performance estimates of 80% sensitivity and 98% specificity, based on prior research describing the discrimination of T3cD from new-onset type 2 diabetes, where, for the combined measurement of adiponectin and interleukin 1 receptor antagonist (IL-1Ra), sensitivity was 83.7% (95% CI, 64.9%-92.0%) and combined measurement of adiponectin and interleukin 1 receptor antagonist specificity was 100.0% (95% CI, 73.6%-100.0%) (Table 1).^16^

To match the assumption that T3cD would be detectable up to 1 year prior to PDAC diagnosis, the cancer-specific biomarker pathway model used performance data for CA19-9 in PDAC samples taken 6 to 12 months before PDAC diagnosis, where the sensitivity for PDAC detection was 32.4% and the specificity for PDAC detection was 95.0%.^23^

The biomarker costs considered in the model ranged between £20 (US $27) and £100 (US $134), with £45 (US $60) used in the base-case scenario. This estimate was informed by the local UK National Healthcare Service (NHS) laboratory cost for the CA 19-9 test, reported at £19.18 (US $25.67).

### Incidence in Target Populations

The T3cD biomarker model included a pancreatogenic diabetes (both PDAC associated and chronic pancreatitis associated) incidence of 10% in the new-onset diabetes cohort.^24^ The cancer-specific biomarker strategy considered a PDAC incidence of 1% among individuals with new-onset diabetes^25^ and 0.02% in the general population.^5^

### Medical Expenditures, Health Utilities, and Population Disease Stage Proportions

Estimated direct medical expenditures for each disease stage were calculated based on 2021-2022 NHS cost collection data.^26^ The model incorporated the estimated costs of diagnostic tests, operation, intensive care stay, physiotherapy, diabetes and dietitian care, pancreatic enzyme replacement, histology services, daily blood tests, endoscopy or interventional radiology services, palliative care, and pancreatic and oncology appointments (eTable in Supplement 1). Systemic chemotherapy was determined by an expert medical oncologist in line with current treatment guidelines.^27^ Chemotherapy costs were extrapolated from recent literature, including gemcitabine monotherapy (£483 [US $647] for 6 cycles),^28^ gemcitabine-capecitabine (£811 [US $1086] for 6 cycles),^28^ and modified FOLFIRINOX (£1360 [US $1820] for 8 cycles).^29^

The model framework assumed fixed health-related quality-of-life utility weights derived using a time trade-off and EuroQol 5-Dimension utility elicitation method. Specific utility weights were assigned to each disease stage (resectable, borderline resectable, locally advanced, and metastatic).^30,31,32^

As part of the clinical impact of introducing the biomarker-based screening strategy, the stated disease stage proportions reflected the anticipated downstaging benefit associated with the biomarker pathway. This included an increase from 10% to 40% in cancers detected at the resectable stage and a reduction of those in the metastatic stage from 55% to 10%, in comparison with standard of care (Table 1).

### Discount and Survival

Costs and future life-years and quality-adjusted life-years (QALYs) were subject to a standard annual discount rate of 3.5%, reflecting the loss in economic value due to a delay in realizing a benefit or incurring a cost associated with treatment.^33^ The decision model included median survival (in years) for each disease stage based on recent literature^2,3,34^ (Table 1).

### Cost-Effectiveness Analyses and Sensitivity Analyses

Analyses were performed from August 2024 to January 2025. The model calculated the net benefits and the incremental cost-effectiveness ratios (ICER) of the difference in costs and QALYs between the biomarker pathways and the standard of care using the following formula: ICER = [total cost (biomarker) − total cost (standard of care)]/[QALY (biomarker) − QALY (standard of care)]. The ICER was compared with a willingness-to-pay (WTP) threshold of £30 000 (US $40 156), as recommended by NICE.^35^

One-way deterministic sensitivity analyses allowed for variation in 1 parameter at a time, ascertaining critical factors for cost-effectiveness. Multiway Monte Carlo probabilistic sensitivity analyses, with 10 000 simulations, allowed for the simultaneous variation of all parameter values. R statistical software, version 4.2.2 (R Project for Statistical Computing) was used.

## Results

### Base-Case Results in the Cancer-Specific Biomarker Strategy vs Standard of Care in New-Onset Diabetes

**Figure 2.**
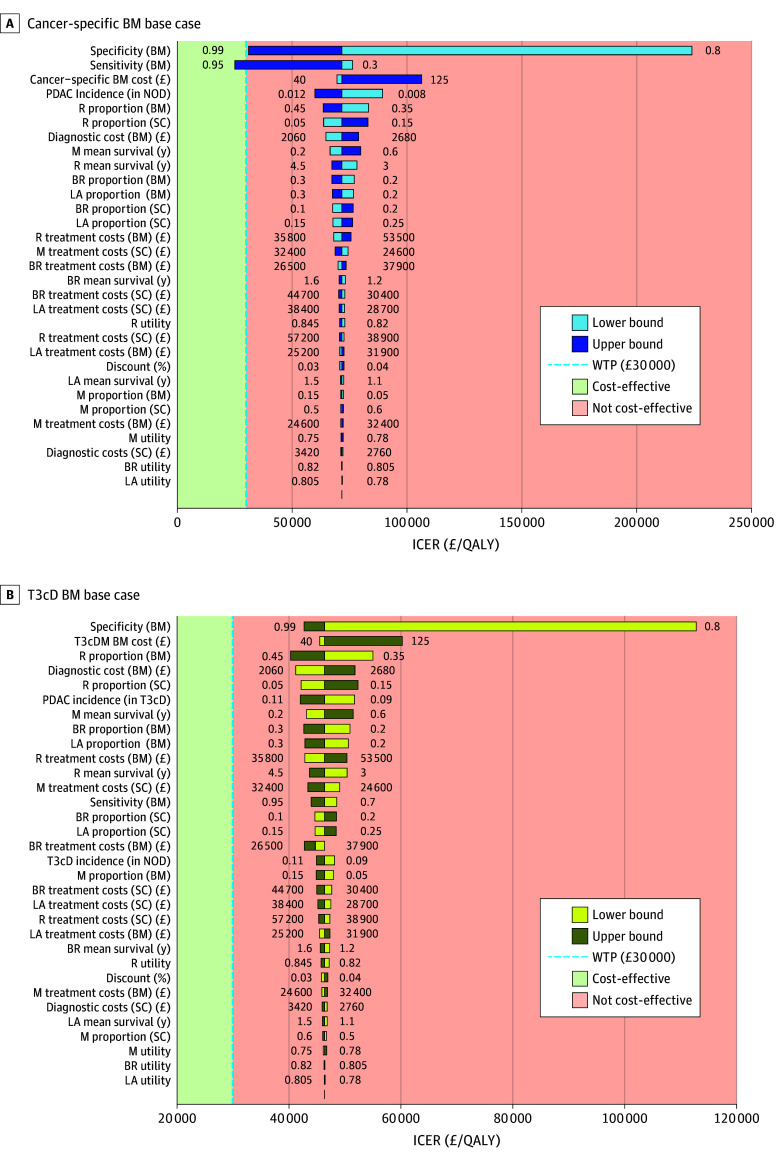
One-Way Sensitivity Analyses in the Cancer-Specific Biomarker (BM) and Type 3c Diabetes (T3cD) BM in New-Onset Diabetes (NOD) A, Cancer-specific BM 1-way sensitivity analysis of incremental cost-effectiveness ratio (ICER). Tornado diagram displaying the key important factors in the cancer-specific BM strategy within the NOD cohort. The base-case scenario did not achieve cost-effectiveness (£71 900 [US $96 241]) at the willingness-to-pay (WTP) threshold of £30 000 (US $40 156). B, T3cD BM 1-way sensitivity analysis. The base-case scenario did not achieve cost-effectiveness (£46 400 [US $62 108]); however, it was closer to the WTP threshold compared with the cancer-specific strategy. The conversion rate was £1 = US $1.34. BR indicates borderline resectable stage; LA, locally advanced stage; M, metastatic stage; PDAC, pancreatic ductal adenocarcinoma; R, resectable stage; QALY, quality-adjusted life-year; and SC, standard of care.

In the base case, detecting PDAC early among individuals with new-onset diabetes using a cancer-specific biomarker test was associated with 0.00271 more life-years and 0.00231 more QALYs, with an additional cost of £165.88 (US $222.14), when compared with the standard-of-care strategy. Considering the chosen WTP threshold of £30 000 (US $40 156), the cancer-specific biomarker test strategy was not cost-effective, with an ICER per QALY of £71 906 (US $96 249). The top 5 factors associated with cost-effectiveness were specificity, sensitivity, cancer-specific biomarker cost, PDAC incidence in new-onset diabetes, and treatment costs of resectable PDAC (Figure 2A). A multiway probabilistic sensitivity analysis of 10 000 simulations demonstrated that 92.3% of case simulations were not cost-effective (eFigure, A, in Supplement 1).

In a simulated population of 200 000 people with new-onset diabetes (the annual incidence of type 2 diabetes in England for 2021-2022 was 243 160^36^), 10 540 people would test positive for the cancer-specific biomarker test, of whom 640 (6.1%) would be true positive, with the remaining 9900 individuals (93.9%) giving false-positive results. Therefore, to detect 1 PDAC case, 16.5 people would need to undergo clinical imaging (Table 2).

**Table 2.** Summary of Base-Case Results for All 3 Biomarker Strategies Compared With the Standard of Care

Characteristic	Biomarker		
	Cancer specific	T3cD specific	Combination
Additional life-years	0.00271	0.00677	0.00217
Additional QALYs	0.00231	0.00577	0.00185
ICER per QALY, £^a^	71 906	46 371	34 223
True-positive cases, No/total No. (%)	640/10 540 (6.2)^b^	16 000/19 600 (81.6)^b^	512/1412 (36.3)^c^
False-positive cases, No/total No. (%)	9900/10 540 (93.9)^b^	3600/19 600 (18.4)^b^	900/1412 (63.7)^c^
No. of people needed to undergo imaging per each positive PDAC case	16.5	12.3	2.4

Abbreviations: ICER, incremental cost-effectiveness ratio; PDAC, pancreatic ductal adenocarcinoma; QALYs, quality-adjusted life-years; T3cD, type-3c diabetes.

^a^ The conversion rate was £1 = US $1.34.

^b^ The true and false positives in the T3cD biomarker and cancer-specific biomarker strategies were calculated in a population of 200 000 people in accordance with the annual incidence of type 2 diabetes in England.

^c^ The true and false positives reported in the combination biomarker pathway were calculated in a smaller population of 19 600 people who tested positive for the T3cD biomarker test applied in 200 000 people with new-onset diabetes.

The cancer-specific biomarker strategy model was also undertaken in the general population, where the incidence of PDAC was 0.02%. This model yielded an ICER per QALY of £3.5 million (US $4.7 million).

### Base-Case Results in the T3cD Biomarker Test Strategy vs Standard of Care in New-Onset Diabetes

The T3cD biomarker test–driven pathway resulted in 0.00677 more life-years and 0.00577 more QALYs compared with the standard-of-care strategy, with additional expenditure of £267.43 (US $357.97) in the T3cD biomarker group. At a PDAC incidence of 1% in the new-onset diabetes cohort, the T3cD biomarker pathway yielded an ICER per QALY of £46 371 (US $62 070), which was above the WTP threshold of £30 000 (US $40 156). One-way deterministic sensitivity analysis demonstrated that the 5 most important factors of cost-effectiveness were T3cD biomarker test specificity, PDAC incidence in T3cD, biomarker cost, proportion of patients diagnosed at resectable stage, and diagnostic test expenditures (Figure 2B). On further multiway probabilistic sensitivity analysis with 10 000 simulations, all cases were above the WTP threshold (eFigure, B, in Supplement 1).

As 1-way analysis showed the T3cD performance to be superior to the cancer-specific test (Figure 2B) and multiway sensitivity analyses showed the cancer-specific test performance to be superior (eFigure, A, in Supplement 1), a direct multiway sensitivity analysis comparing the 2 biomarker types was undertaken. This analysis revealed that 55.6% of T3cD biomarker test simulations were cost-effective compared with 44.4% of the cancer-specific biomarker simulations (eFigure, C, in Supplement 1).

In a simulated population of 200 000 people with new-onset diabetes, application of the T3cD biomarker test would lead to 1.3 people being scanned to detect 1 pancreatogenic disease case (including chronic pancreatitis and PDAC) and 12.3 people being scanned to detect 1 PDAC case. Of 19 600 individuals with a positive T3cD biomarker test, 16 000 (81.6%) would be true-positive T3cD cases and 3600 (18.4%) would be false-positive T3cD cases (Table 2).

### Base-Case Results in the Combination Biomarker Strategy (T3cD and Cancer Specific) vs Standard of Care in New-Onset Diabetes

**Figure 3.**
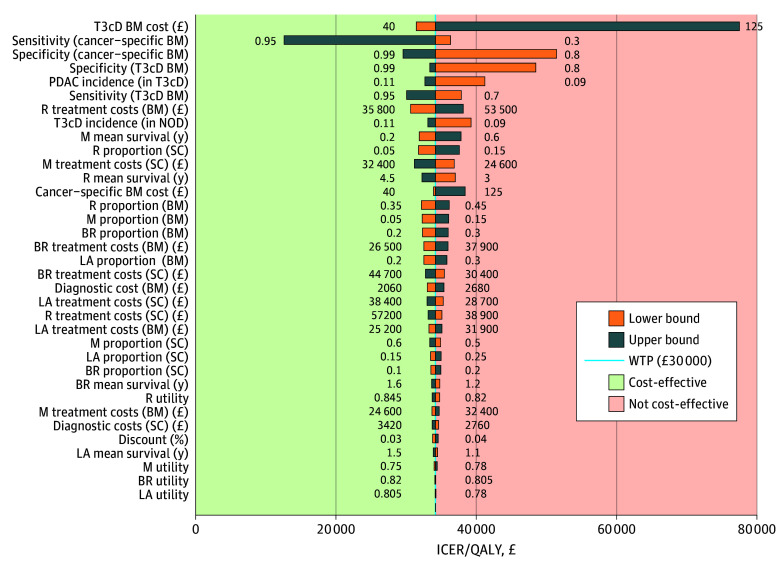
One-Way Sensitivity Analyses in the Combination of Cancer-Specific and Type 3c Diabetes (T3cD) Biomarker (BM)–Associated Screening in New-Onset Diabetes (NOD) Combined (cancer-specific and T3cD) BM 1-way sensitivity analysis. The base-case scenario was close to the willingness-to-pay (WTP) threshold at £34 200 (US $45 778). The conversion rate was £1 = US $1.34. BR indicates borderline resectable stage; ICER, incremental cost-effectiveness ratio; LA, locally advanced stage; M, metastatic stage; PDAC, pancreatic ductal adenocarcinoma; R, resectable stage; QALY, quality-adjusted life-year; and SC, standard of care.

In the combined biomarker test pathway, 0.00217 more life-years and 0.00185 more QALYs were gained with an additional cost of £63.16 (US $84.54) compared with the standard-of-care strategy. At the WTP threshold of £30 000 (US $40 156), the combined biomarker strategy approached cost-effectiveness at an ICER per QALY of £34 223 (US $45 809). The most important parameters for cost-effectiveness were T3cD biomarker cost, cancer-specific biomarker test sensitivity and specificity, T3cD biomarker test specificity, and PDAC incidence in the T3cD cohort (Figure 3). A multiway probabilistic sensitivity analysis revealed that 26% of simulated cases were cost-effective (eFigure, D, in Supplement 1).

Biomarker sensitivity and specificity appeared to be highly important in all 1-way sensitivity analyses. Hence, for the combination biomarker pathway, a 2-way sensitivity analysis was undertaken that allowed only for variation of sensitivity and specificity of the cancer-specific biomarker. In addition, the direct interaction between sensitivity and specificity is illustrated in eFigure, E, in Supplement 1, demonstrating the cancer-specific biomarker performance level at which the WTP threshold is met. There is a large region of cost-effective sensitivity-specificity combinations, providing that the base-case parameters remain fixed. When the combination biomarker pathway was used in a simulated population of 200 000 people with new-onset diabetes, 2.4 scans were performed to detect 1 PDAC case (Table 2).

## Discussion

We report that enriching the high-risk group of individuals with new-onset diabetes for PDAC using a primary T3cD biomarker test followed by a secondary PDAC-specific test was more cost-effective than the sole use of either biomarker test. Across all strategies, one of the most important cost-effectiveness parameters was biomarker specificity. Future research should focus on developing biomarkers with sufficient specificity to support cost-effective screening strategies.

The development of early PDAC-specific biomarker signatures, regardless of their composition, faces multiple challenges. Although many candidates have been proposed,^37^ few have been taken through formal preclinical validation. A key barrier is the lack of well-annotated, prediagnostic PDAC samples, limiting insight into biomarker signatures in the months or years prior to PDAC diagnosis. Exceptions include CA19-9, which has been widely investigated in blood and urine samples using prediagnostic PDAC cohorts.^23,38,39^ Its prediagnostic performance characteristics were chosen for this study. However, these cohorts were not designed for the development of PDAC surveillance or for the screening and lack the critical pancreatic cancer–specific annotation required for this purpose.

Targeting high-risk groups offers an alternative approach to population-wide biomarker-associated screening. The new-onset diabetes cohorts of the Chronic Pancreatitis, Diabetes and Pancreatic Cancer Consortium^40^ and UK-EDI^17^ studies will allow for the prediagnostic performance of future biomarker tests to be determined. There are currently very few studies reporting biomarkers or tests with the potential to distinguish T3cD from new-onset type 2 diabetes,^16,41^ highlighting the need for further research.

The application of either T3cD-specific or cancer-specific biomarker screening in new-onset diabetes would capture only patients with PDAC and diabetes, but it would not facilitate detection of individuals with PDAC who do not have diabetes. The proportion of PDAC cases with new-onset diabetes is 25.1% to 34.9%,^42,43^ which is significantly higher than the proportion of cases attributed to familial PDAC (10%), for which screening or surveillance exists. Compared with the high-risk familial PDAC group, in which recommendations include lifelong screening of patients with an estimated lifetime risk greater than 10%,^44^ in the new-onset diabetes group, screening would be required for a shorter period of approximately 3 years after diabetes diagnosis, as thereafter the PDAC risk decreases from 6-fold to 8-fold to 1.5-fold.^45^ Given the low incidence of PDAC and its extremely high mortality, taking a pragmatic approach and implementing screening among individuals with new-onset diabetes, although imperfect, would be a vital step in improving outcomes.

Although not included in our cost-effectiveness analysis, incidental findings resulting from imaging warrant consideration. A recent prospective pilot study, assessing the feasibility of cross-sectional imaging for PDAC screening in individuals with new-onset diabetes, demonstrated that of 93 scanned participants, only 1 had PDAC, 55.9% (52 of 93) had extrapancreatic findings, and 12.9% (12 of 93) had additional pancreatic incidental findings.^46^ Our sequential biomarker strategy resulted in the fewest imaging events per PDAC case. Furthermore, almost every individual undergoing imaging on the T3cD test–driven screening pathway had pancreatic pathologic findings (1.3 people scanned per T3cD case). The likely additional health care and cost benefits resulting from identifying chronic pancreatitis earlier (73% of T3cD-positive cases^15^), including addressing alcohol cessation and nutritional deficiencies among these individuals, were not considered here.

### Limitations

This study has some limitations. T3cD biomarker sensitivity and specificity were based on adiponectin and IL1-Ra performance in diagnostic samples.^16^ However, IL1-Ra distinguished PDAC cases from healthy controls up to 1 year before cancer diagnosis.^16^ As T3cD occurs early in PDAC development, with elevation in hemoglobin A_1c_ levels up to 36 months prior to cancer diagnosis, the expectation is that a diagnostic T3cD biomarker may have a similar or slightly lower performance in preclinical samples.^47^

Screening is envisioned in primary care, where patients older than 50 years with a diabetes diagnosis undergo a T3cD biomarker test. A positive result prompts a cancer-specific biomarker test, followed by referral to secondary care if the biomarker test results are positive. Clinical implementation depends on ongoing development and preclinical evaluation of evidence-based biomarkers to identify individuals with new-onset diabetes at highest risk for PDAC.

This analysis did not consider the costs and benefits associated with diagnosing and treating chronic pancreatitis in T3cD-positive individuals, as this was not our primary focus. Furthermore, only direct medical costs were included. We recognize that incorporating indirect costs could provide a more comprehensive societal perspective and potentially influence the cost-effectiveness outcomes.

## Conclusions

This economic evaluation suggests that using a T3cD biomarker to generate an enriched group of individuals with new-onset diabetes at higher risk of PDAC, followed by a cancer-specific biomarker, offers a near cost-effective strategy for early PDAC detection. Our findings can be used to inform biomarker-associated early detection strategies for PDAC.

## Notes

Supplement 1. **eTable.** Direct Medical Expenditures Utilised for Pancreatic Cancer Diagnosis/Treatment Cost Calculations **eFigure.** Multiway and 2-Way Sensitivity Analyses in the Cancer-Specific Biomarker (BM), the Type 3c Diabetes Mellitus (T3cDM) BM, and the Combination (T3cDM and Cancer-Specific) BM-Associated Screening in New-Onset Diabetes

Supplement 2.
Data Sharing Statement
