# Supplementary material for: Cost-Effectiveness of Biomarker-Associated Early Pancreatic Cancer Detection in New-Onset Diabetes
Source: JAMA Netw Open. 2025 Oct 17;8(10):e2538031. doi: 10.1001/jamanetworkopen.2025.38031 (PMC12534857; doi:10.1001/jamanetworkopen.2025.38031)
Supplement: Supplement 1. — eTable. Direct Medical Expenditures Utilised for Pancreatic Cancer Diagnosis/Treatment Cost Calculations eFigure. Multiway and 2-Way Sensitivity Analyses in the Cancer-Specific Biomarker (BM), the Type 3c Diabetes Mellitus (T3cDM) BM, and the Combination (T3cDM and Cancer-Specific) BM-Associated Screening in New-Onset Diabetes [file jamanetwopen-e2538031-s001.pdf]

Supplementary Online Content

Stefanova I, Thompson N, Oldfield L, et al. Cost-effectiveness of biomarker-associated early pancreatic cancer detection in new-onset diabetes. *JAMA Netw Open.* 2025;8(10):e2538031. doi:10.1001/jamanetworkopen.2025.38031

eTable. Direct Medical Expenditures Utilised for Pancreatic Cancer Diagnosis/Treatment Cost Calculations

eFigure. Multiway and 2-Way Sensitivity Analyses in the Cancer-Specific Biomarker (BM), the Type 3c Diabetes Mellitus (T3cDM) BM, and the Combination (T3cDM and Cancer-Specific) BM-Associated Screening in New-Onset Diabetes

This supplementary material has been provided by the authors to give readers additional information about their work.

© 2025 Stefanova I et al. *JAMA Network Open.*

eTable. Direct Medical Expenditures Utilised for Pancreatic Cancer Diagnosis/Treatment Cost Calculations

**Medical test / treatment / service**	**Cost**
CT TAP scan	£146.34
EUS and biopsy	£2,882.75
PET	£935.58
Pancreaticoduodenectomy	£8199.64 - £15,687.25
Intensive care costs (24hrs)	£1668.02 - £1,844.77
Creon (2-week supply)	£28.25
Diabetes care	£140.5 - 273.3
Physiotherapy	£73.14
Pancreas multi-disciplinary meeting	£154.67
Outpatient Pancreas Surgery appointment 1st	£269.25
Outpatient Pancreas Surgery appointment follow-up	£255.99
Outpatient Oncology appointment 1st	£206.47
Outpatient Oncology appointment follow-up	£164.19
Pancreas Specialist Nurse appointment 1st	£216.24
Pancreas Specialist Nurse appointment follow-up	£176.46
Daily Integrated Bloods Service	£2.39
Palliative care inpatient	£596.25
Palliative care outpatient	£226.92
Dietitian appointment 1st	£134.02
Dietitian appointment follow-up	£152.14
ERCP and stent	£4648.47 - £7,001.47
PTC and metal stent	£3,889.60
Percutaneous liver biopsy	£1,780.46

CT TAP – computed tomography thorax, abdomen and pelvis; EUS – endoscopic ultrasound; PET – positron emission tomography; ERCP – endoscopic retrograde cholangiopancreatography; PTC – percutaneous transhepatic cholangiography

© 2025 Stefanova I et al. *JAMA Network Open.*

eFigure. Multiway and 2-Way Sensitivity Analyses in the Cancer-Specific Biomarker (BM), the Type 3c Diabetes Mellitus (T3cDM) BM, and the Combination (T3cDM and Cancer-Specific) BM-Associated Screening in New-Onset Diabetes

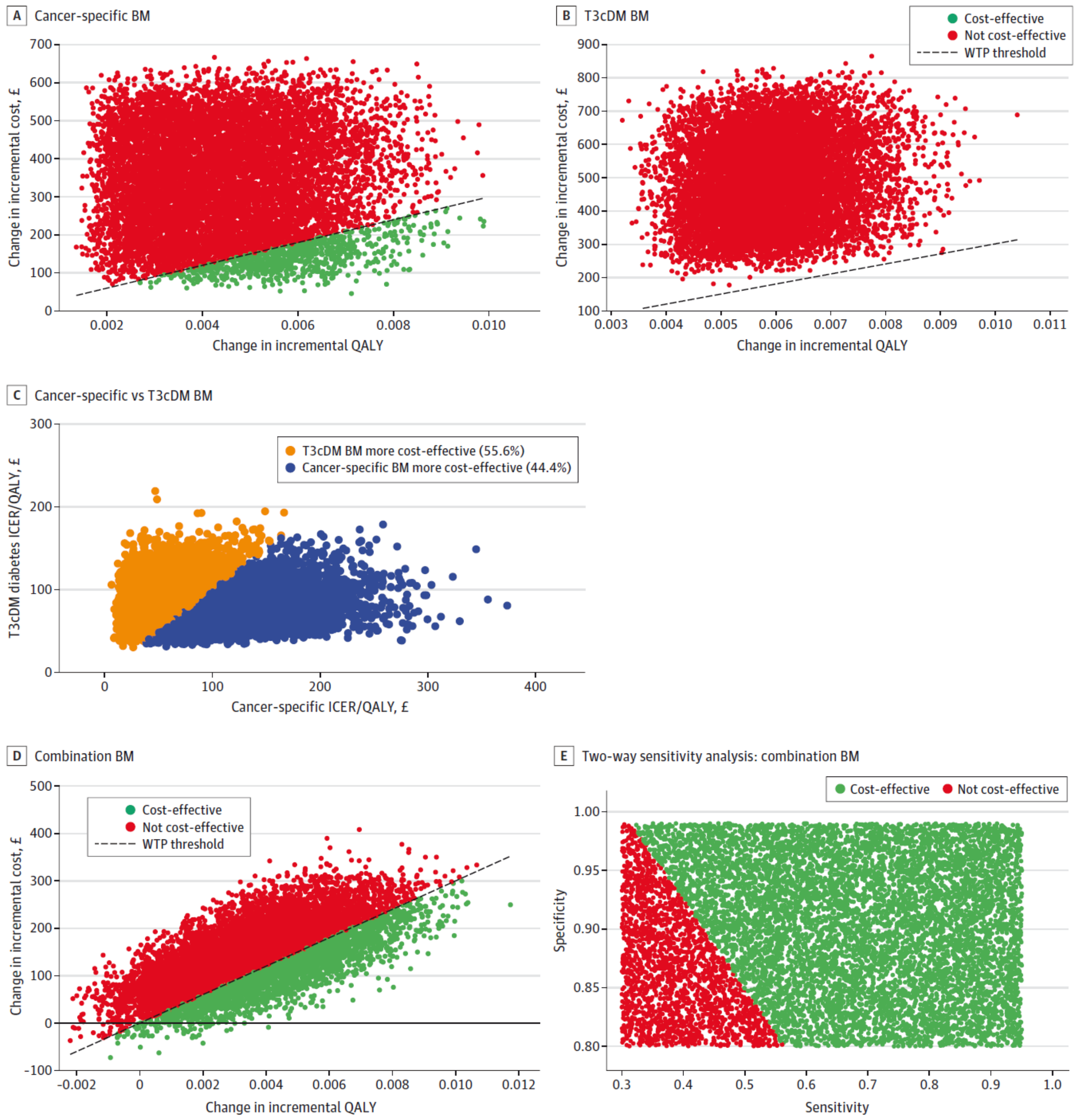

A, Multiway Monte Carlo probabilistic sensitivity analysis in the cancer-specific BM strategy allowing for variation of all parameters. Most case simulations are above the willingness-to-pay (WTP) threshold. B, Multiway probabilistic sensitivity analysis in the T3cDM BM strategy. All case simulations are above the WTP threshold. C, A comparison of a cancer-specific BM vs a T3cDM BM, illustrating which scenario is more cost-effective over 10 000 simulations. D,

© 2025 Stefanova I et al. *JAMA Network Open.*

Multiway probabilistic sensitivity analysis in the combination (T3cDM and cancer-specific) BM strategy. E, Two-way sensitivity analysis allowing variation of only sensitivity and specificity of the cancer-specific BM in the combination BM strategy. ICER indicates incremental cost effectiveness ratio; and QALY, quality-adjusted life-yeary.

© 2025 Stefanova I et al. *JAMA Network Open.*
