# Supplementary material for: Cost-Effectiveness of Biomarker-Associated Early Pancreatic Cancer Detection in New-Onset Diabetes
Source: JAMA Netw Open. 2025 Oct 17;8(10):e2538031. doi: 10.1001/jamanetworkopen.2025.38031 (PMC12534857; doi:10.1001/jamanetworkopen.2025.38031)
Supplement: Supplement 2. — Data Sharing Statement [file jamanetwopen-e2538031-s002.pdf]

Data Sharing Statement

Stefanova. Cost-Effectiveness of Biomarker-Associated Early Pancreatic Cancer Detection in New-Onset Diabetes. *JAMA Netw Open*. Published October 17, 2025. doi:10.1001/jamanetworkopen.2025.38031

Data

Data available: Yes
Data types: Data (not involving human participants)
How to access data: irena.stefanova@liverpool.ac.uk
When available: beginning date: 04-04-2025, end date: 04-04-2030

Supporting Documents

Document types: Statistical/analytic code
How to access documents: irena.stefanova@liverpool.ac.uk
When available: beginning date: 04-04-2025, end date: 04-04-2030

Additional Information

Who can access the data: Data will be available to any researchers upon a reasonable request.
Types of analyses: Markov State Transition Model and sensitivity analyses.
Mechanisms of data availability: After approval of request.
